# MIDLIFE MEDIATORS OF EDUCATIONAL DIFFERENCES IN DEMENTIA RISK IN LATER LIFE:PRELIMINARY RESULTS FROM THE HUNT STUDY

**DOI:** 10.1093/geroni/igad104.3468

**Published:** 2023-12-21

**Authors:** Teferi Mekonnen, Vegard Skirbekk, Hans-Peter Kohler, Yaakov Stern, Asta Håberg, Bo Engdahl, Ekaterina Zotcheva, Bjorn Heine Strand

**Affiliations:** Norwegian Institute of Public Health, Oslo, Oslo, Norway; Norwegian Institute of Public Health, Oslo, Oslo, Norway; University of Pennsylvania, University of Pennsylvania, Pennsylvania, United States; Columbia University, New York City, New York, United States; Norwegian University of Science and Technology, Trondheim, Sor-Trondelag, Norway; Norwegian Institute of Public Health, Oslo, Oslo, Norway; Norwegian National Centre for Ageing and Health, Tønsberg, Vestfold, Norway; Norwegian Institute of Public Health, Oslo, Oslo, Norway

## Abstract

There is a well-known association between education and dementia risk. However, there is limited knowledge of the mediating mechanisms. Using data from the HUNT study linked with registries (n=9745), we examined the mediating role of occupation complexity, smoking, physical inactivity, systolic blood pressure, body mass index(BMI), cardiovascular diseases, hearing impairment, diabetes, and depression score (only in HUNT2) assessed during HUNT1(age 45years) and HUNT2(age 56years) for the association between education and dementia among 70+ years using causal mediation analysis. Among eligible participants, 15.7% had dementia at age 70+ years, completed compulsory education (25.7%), lower-secondary (41.7%), upper-secondary(12%), undergraduate(16.5%), and postgraduate education(4%). For every one-unit increase in educational level from compulsory education to the following levels, a decrease in the odds of having dementia among 70+ years was found (OR=0.74). Midlife mediators assessed at 45 and 56 years jointly explained 17.3% and 26.8% of the effect of education on dementia among 70+ years, respectively. Smoking and systolic blood pressure (each explained 3.7%) followed by BMI(explained 2.3%) showed a significant independent mediating role for the educational differences in dementia among 70+ years. Whereas, among the mediators assessed at the age of 56 years, systolic blood pressure(explained 6.1%), hearing impairment (explained 4.4%), smoking(explained 3.1%) and depression score(explained 2.7%) showed significant independent mediating effects. Midlife risk factors of dementia considered in this study explained nearly one-fourth of the effect of education on dementia in particular, systolic blood pressure, hearing impairment, smoking, depression score and BMI. If truly causal, these factors could indicate potential targets for interventions.

